# No Selective Attentional Shift despite Prefrontal Activation during a Working-Memory Task with Unconscious Stimuli

**DOI:** 10.1523/ENEURO.0183-25.2025

**Published:** 2025-09-30

**Authors:** Tiziana Pedale, Olympia Karampela, Johan Eriksson

**Affiliations:** ^1^Umeå Center for Functional Brain Imaging and Department of Medical and Translational Biology, Umeå University, Umeå 901 87, Sweden; ^2^Department of Psychology, Umeå University, Umeå 901 87, Sweden

**Keywords:** attention, consciousness, endogenous control, fMRI, frontoparietal network, working-memory

## Abstract

A key process for successful working memory is to prioritize task-relevant information over distraction, i.e., to control attentional deployment. Here we investigate to what extent attentional control during a delayed match-to-sample task can be achieved when to-be-remembered items were presented unconsciously together with distracting information and with a prestimulus cue that indicated whether the target was likely to appear on the left or right side of the screen. This expectation was sometimes violated (20% of trials), requiring reorienting of attention to successfully solve the task. Moreover, the cue was uninformative of the exact location of the target, which could appear on the top, middle, or bottom part of the screen. Participants performed better than chance on unconscious trials only when the cue correctly indicated target side, suggesting an inability to reorient attention when the cues were invalid. Neural activity (fMRI BOLD signal change) in medial and lateral prefrontal cortex was significant for unconscious valid-cue trials and remained significant for invalid-cue trials only in the lateral prefrontal cortex, although neither region was significantly modulated by cue validity. Parietal regions did not show significant activation for valid or invalid unconscious stimuli. Thus, even though activity in brain regions associated with cognitive control reached significant levels for unconscious stimuli, there was no evidence for adaptive deployment of selective attention based on unconscious information, which was the case for conscious stimuli. The ability to control attentional deployment appears to differ between conscious and unconscious working memory.

## Significance Statement

A key process for successful working memory is controlling attentional deployment. Here, we investigated how attention is controlled during a working-memory task with unconscious stimuli, by testing participants’ ability to shift attention from an expected target location to an alternative location when necessary. Despite prefrontal cortex activation, participants did not reveal shifts of attention when cues were invalid, suggesting an inability to adaptively deploy selective attention based on unconscious information. Additionally, no activation was observed in parietal attentional areas for either valid or invalid unconscious stimuli. These results indicate that, unlike conscious working memory, unconscious stimuli do not trigger flexible attentional shifts, highlighting a key distinction in how attention is controlled based on awareness.

## Introduction

Although working memory is often thought of as a conscious process, more than a decade of research has demonstrated that also unconsciously presented task-relevant information can be stored in the short term [see Gambarota et al. (2022) for a meta-analysis]. However, working memory as a concept goes beyond simple short-term maintenance of information and also involves various control processes [see Eriksson et al. (2015) for overview]. For example, prioritization of task-relevant information over distraction is crucial for effective working-memory functioning, such that unnecessary storage of distracting items is a hallmark of lower working-memory capacity (Vogel et al., 2005; McNab and Klingberg, 2008; Fukuda and Vogel, 2009). To what extent executive components of working memory can also be implemented in relation to unconscious information is less well understood. Although most previous studies have been done outside the context of working-memory tasks, there is a relevant body of research on the topic of unconscious cognitive control, with mixed findings [see van Gaal et al. (2012) for review]. Response inhibition (van Gaal et al., 2008, 2010) and conflict detection/resolution (Ursu et al., 2009; D'Ostilio and Garraux, 2012; Desender et al., 2016) triggered by unconsciously presented stimuli alters response time and increases neural activity in the dorsomedial prefrontal cortex (dmPFC), a core hub for regulating cognitive control (Shenhav et al., 2016). In contrast, Dehaene et al. found no modulation of dmPFC from congruent/incongruent masked primes (Dehaene et al., 2003). Indeed, masked primes engaged the dmPFC for both congruent and incongruent items, whereas conscious primes only engaged the dmPFC if they were incongruent. Using electroencephalography to measure event-related potentials, Pavone et al. demonstrated that an error-related negativity, indicating response monitoring, was elicited also by unconscious stimuli (Pavone et al., 2009). In contrast, Woodman did not find a significant error-related negativity for unconscious stimuli, despite an attentional shift (Woodman, 2010). On the other hand, Cohen et al. demonstrated increased connectivity between dmPFC and occipital cortex for incorrect responses to both conscious and unconscious stimuli (Cohen et al., 2009).

Taken together, these prior findings show that unconsciously presented stimuli can alter neural activity in the dmPFC, indicative of flexible control processing, though results vary across studies. Although suggestive that such control processes could potentially be utilized in the context of a working-memory task, research on this aspect of unconscious working memory remains limited. In a recent set of experiments, we investigated the storage and purposeful use of unconscious information in a visual working-memory task with conscious and unconscious stimuli (Pedale et al., 2023). During fMRI scanning, participants performed a delayed match-to-sample task in which task relevance of two competing stimuli was indicated by a cue shape. Multivariate pattern analysis revealed that for conscious trials, only the target was retained during the delay phase, indicating successful attentional filtering. On the contrary, both target and distractor appeared to be stored when presented unconsciously. Importantly, task relevance was nevertheless encoded also for unconscious stimuli, as demonstrated by better-than-chance decoding of task role during the test phase. These results indicate that when task relevance is modulated by perceptual features (i.e., a shape), participants can differentiate between relevant and distracting information also for unconscious stimuli but that the attentional control of such information differs for conscious compared with unconscious stimuli. We here aim to further investigate how spatial attention is controlled during a working-memory task with unconscious stimuli, by testing participants’ ability to shift attention from an expected target location to an alternative location when required to solve the task. The present study builds directly on this prior work by using a similar design as Pedale et al. (2023). Here, participants are again instructed to remember a target shape while ignoring a distractor shape. Critically, we introduce a spatial cue that probabilistically predicts the most likely location of the target shape. Reorienting of attention is required during invalid trials, in which the target appears at a noncued location, requiring participants to shift attention away from the expected location. Previous studies on working memory have highlighted the critical role of cue validity in guiding attention (Vossel et al., 2006, 2012; Corbetta and Shulman, 2011), and our goal here is to extend this research into the unconscious domain. By focusing on conditions that require flexible attentional reallocation, we aim to better understand the extent to which adaptive deployment of spatial attention is preserved with unconscious stimuli.

## Materials and Methods

### Participants

A convenience sample of 33 participants were initially recruited at the Umeå University campus. Of these, six were excluded: two for failing to complete the experiment, two for failing to follow task instructions, and two for MRI data issues. The final sample thus consisted of 27 participants (14 female; mean age, 23.4 years; range, 18–33). All participants had normal or corrected to normal vision, were right eye-dominant, were right-handed, and reported not to be affected by colorblindness or any neurological or psychiatric disease. They gave written informed consent and were paid 500 SEK for participation. The study was approved by the Swedish Ethical Review Authority (2019-04565).

### Experimental design

The experiment was implemented in E-Prime 2.0. Participation consisted of attending two occasions within a 7 d interval: training and the actual experiment. The training was constituted by a shorter version of the task with the purpose to ensure that each participant understood the task and that the continuous flash suppression (CFS) manipulation worked as intended (see below). The actual experiment consisted of 300 delayed match-to-sample trials, divided into six blocks (50 trials each).

Each trial was drawn randomly from the five conditions and began with an intertrial interval (randomly between 3 and 9 s) before the cue presentation. The cue was either a circle or a diamond shape presented in the center of the screen for 1.75 s on a green background and indicated which shape was relevant on a trial-by-trial basis. At the center of the cue, a letter (“L” and “R” for left and right, respectively) indicated whether the target was likely to appear on the left or right side of the screen. Immediately after the cue presentation, a gray screen was displayed (0.25 s), followed by the sample presentation (1 s). Participants were instructed to keep their gaze on the central white fixation cross during the sample presentation. The sample consisted of a gray silhouette of the cue shape (the target) presented together with a nonrelevant shape (the distractor). Thus, if the circle was the defined target, the diamond acted as a distractor for that trial, and vice versa. The target side expectation, indicated by the cue, was violated in ∼20% of trials. The task, thus, consisted of the following five conditions: 54 conscious trial with a valid cue (i.e., the target appeared on the side indicated by the cue), 24 conscious with an invalid cue (i.e., the target appeared on the opposite side indicated by the cue), 126 unconscious with a valid cue, 54 unconscious with an invalid cue, and 42 absent trials. The target was presented in an equal number of trials in each of the six possible positions (up, middle, down, on either the left or right side of the screen) and the task consisted of remembering only its vertical position. The distractor could appear in one of the three vertical positions on the opposite side (right or left), but never in the same vertical position as the target. For conscious trials only, the remaining four positions not occupied by either the target or distractor were filled with placeholder figures.

We used CFS to manipulate the visual experience of the memory sample. A mirror stereoscope was used to isolate visual input from one side of the screen to the participant's corresponding eye. In the conscious condition, the sample was presented to the dominant (right) eye, and the gray shapes (RGB, i.e., red–green–blue = 198, 198, and 198) were superimposed on colored squares of random composition (Mondrians) that were flashed (10 Hz) to the same eye. In the unconscious condition, the sample consisted of the gray shapes on a gray background (RGB = 210, 210, and 210) presented only to the nondominant (left) eye, whereas Mondrians were flashed to the dominant eye. This procedure suppressed the sample from conscious experience (Tsuchiya and Koch, 2005). During the unconscious trials, the sample was presented for 500 ms, whereas the Mondrians were flashed for 1 s to minimize the risk of adaptation after-effects (Tsuchiya and Koch, 2005). During the absent trials, Mondrians were presented to the dominant eye whereas an empty gray background (RGB = 210, 210, and 210) was presented to the nondominant eye. Importantly, unless the sample stimuli broke suppression, unconscious and absent trials led to the same visual experience (experiencing only Mondrians). These “absent” trials served as a reference condition for the unconscious trials, such that the subjective visual experience was identical for unconscious and absent trials (seeing only Mondrians), and any response bias could be estimated independently from target and distractor effects (i.e., the tendency to respond “no” more often than “yes” when no target and distractor was seen).

After a delay phase (3–5 s) consisting of a gray screen with a central white dot, a memory probe was presented. The probe consisted of an indicator line placed either at the top, middle, or bottom position of possible target/distractor positions. The task consisted of recognizing if the probe was placed at the same vertical level as the target or not (yes/no response). If participants did not visually experience a target stimulus (i.e., only experienced Mondrians), they were instructed to guess according to their gut feeling/first alternative that came to mind at the appearance of the probe. The participants had a maximum time of 5 s to answer, after which the next trial was initiated. The chance for the probe to point to the target position was 50%. For the conscious and unconscious trials, the probe pointed to the correct target location (“target-match”) or the distractor location (“distractor-match”), but never to an empty position. Thus, for conscious trials, there were 39 “target-match” probes (27 valid, 12 invalid) and 39 “distractor-match” probes (27 valid, 12 invalid). For unconscious trials, there were 90 “target-match” probes (63 valid, 27 invalid) and 90 “distractor-match” probes (63 valid, 27 invalid).

**Figure 1.**
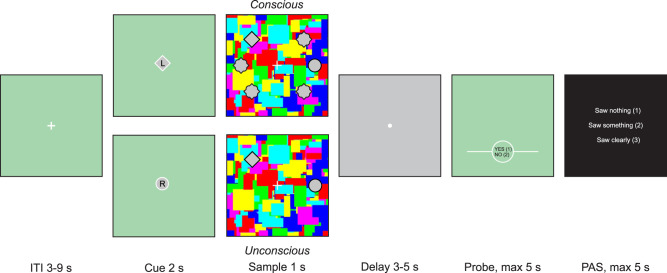
Trial structure. After each intertrial interval (ITI) a cue was presented (2 s = 1.75 s cue + 0.25 s gray screen), informing participants of which shape was the target on this specific trial (diamond or circle) and whether the target was expected to appear most likely on the left or right side of the screen (L/R). The target to be remembered as well as the distractor was then presented in the context of continuous flash suppression (see Materials and Methods). For conscious trials (top part of “sample”), all possible positions that were not occupied by the target or distractor were filled with placeholders, while these placeholders were absent for unconscious and “absent” trials. Note that the target/distractor/placeholders are here outlined in black only for illustrative purposes; in the actual experiment they consisted of homogenous gray shapes with no outline. Participants were instructed to remember the vertical position of the target and answer yes/no whether or not the probe pointed to the correct vertical position. After each trial participants reported whether or not they had seen any stimulus during the sample presentation, using a 3-point perceptual awareness scale (PAS).

At the end of each trial, participants were asked to report their perception of the sample on a modified (3-point) perceptual awareness scale (PAS; Ramsoy and Overgaard, 2004), ranging from no perceptual experience (PAS = 1), vague perceptual experience (PAS = 2), and clear perceptual experience (PAS = 3; for a similar approach, see Christensen et al., 2006; Bergstrom and Eriksson, 2018; Fontan et al., 2021; Pedale et al., 2023). The PAS prompt was presented for a maximum of 5 s. See Figure 1 for illustration of the task. The contrast of the sample shapes during the nonconscious trials was adjusted every 10 nonconscious trials based on the PAS response, ensuring that participants did not break the suppression for at least 80% of unconscious trials. Specifically, if the participants reported some experience of the shapes (PAS > 1) => 3 out of 10 unconscious trials, the contrast between the shapes and the background decreased (thus making the processing of the unconscious stimuli harder); otherwise, it increased (thus making the processing of the shapes easier). This adjustment was done to maximize the generally weak processing of the unconscious stimuli. Notably, this contrast adjustment procedure is independent of task performance, since the adjustment is based solely on the visibility ratings. Each contrast value consisted of a 2-point increase/decrease in RGB value of the gray shapes (range, 206–182) relative to the gray background (RGB = 210, 210, and 210). Each participant started the training with a contrast value of RGB = 196, 196, and 196 and started each subsequent block with the last contrast value reached in the previous block.

### MRI parameters

The MRI data were collected with a GE 3 T Discovery MR750 scanner (32-channel receive-only head coil). Each participant underwent one fMRI session with six functional runs (410 volumes each) of scanning using a T2*-weighted gradient echo pulse sequence; echoplanar imaging; field of view, 25 cm; matrix size, 96 × 96; slice thickness, 3.4 mm; 37 slices with 0.5 mm interslice skip; and an ASSET acceleration factor of 2. The volumes covered the whole cerebrum and most of the cerebellum. The acquisition orientation was oblique axial and aligned with the anterior and posterior commissures, and the slices were acquired in interleaved order with time echo (TE), 30 ms; time repetition (TR), 2 s; and flip angle, 80°. Between the third and fourth functional runs, a high-resolution T1-weighted structural image was collected with Fast Spoiled Gradient Echo (FSPGR) with TE, 3.2 ms; TR, 8.2 ms; TI, 450 ms; and flip angle, 12°.

### Data analysis

#### Behavioral statistical analysis

Only trials in absent and nonconscious conditions with PAS = 1 and trials with PAS = 3 in the conscious condition were included in the behavioral data analyses. For the analyses of accuracy, a hit was defined as a “yes” response to a “target-match” probe, whereas a “yes” response to a “distractor-match” probe was defined as a false alarm. Having computed the hits and false alarms, we calculated for each condition a sensitivity measure, *d*’, according to the formula: *d*’ = *z* (hits rate) − *z* (false alarm rate) (Stanislaw and Todorov, 1999).

All statistical comparisons were conducted using one-tailed *t* tests. Specifically, for the *d*’ score, we performed one-tailed one-sample *t* test since we wanted to evaluate whether performance was significantly above chance (i.e., >0). For comparisons between valid and invalid trials, we used one-tailed two-sample *t* test because we expected higher performance (i.e., higher *d*’ score) for validly cued trials than invalid ones. Similarly, for reaction times, we used one-tailed two-sample *t* test since we predicted faster responses for validly cued trials compared with invalid trials.

To further substantiate any null result, we also report the Bayes factor (BF_01_) according to the following interpretation: BF_01_ < 1, no evidence; 1–3, anecdotal evidence; 3–10, substantial evidence; 10–30, strong evidence; 30–100, very strong evidence; and >100, decisive evidence (Keysers et al., 2020).

#### Preprocessing and fMRI data analysis

Image preprocessing and intrasubject modeling was conducted with SPM12 (Wellcome Department of Imaging Neuroscience) running on Matlab 8.4 (MathWorks) using custom-made Matlab scripts for batching. All images were (1) slice time corrected using the first slice as a reference, (2) corrected for head movements between image volumes, (3) unwarped to remove residual movement-related variance, and (4) coregistered to high-resolution structural data. The structural images were normalized to the Montreal Neurological Institute template using DARTEL (Ashburner, 2007), and the resulting parameters were used for normalization of the functional images, which were resampled to 2 mm isotropic voxel size. Finally, the functional images were smoothed with an 8 mm FWHM Gaussian kernel for the univariate analyses.

For intrasubject modeling, a general linear model (GLM) was used. The model consisted of the following regressors of interest: valid and invalid conscious with PAS = 3, valid and invalid unconscious with PAS = 1, and absent with PAS = 1, for the sample and probe trial phases. The regressors of interest related to the probe phase were divided by the response category (hits and misses for “target-match,” false alarms, and correct rejections for “distractor-match” probes). The regressors related to the sample and probe phase was modeled with zero duration. The model also included the following nuisance regressors: irrelevant presentation conditions (conscious with PAS < 3, nonconscious with PAS > 1, and absent with PAS > 1) and missed responses by trial phase (sample and probe). Head motion (six parameters) was included as covariates of no interest. All regressors except for head motion were convolved with the SPM12 canonical hemodynamic response function. A high-pass filter (cutoff, 128 s) was applied to remove low-frequency drift in the data and the autocorrelation model was global AR(1).

Data were analyzed using a two-stage summary statistics random-effects model (Friston et al., 1995). Contrast maps were computed on beta maps resulting from the estimated first-level GLMs to reveal for conscious and nonconscious conditions, brain regions subtending visuospatial processing during the sample presentation. Individuals’ maps subtending conscious and nonconscious visuospatial networks were taken to second-level random-effects analyses (one-sample *t* tests) to account for inter-individual variability. Multiple-comparisons correction of statistical maps at the second level was conducted on the whole brain using cluster-based extent thresholding of *p* < 0.05 [family wise error (FWE) corrected] calculated based on the Gaussian random field method and following cluster-defining threshold of *p* < 0.001.

We used the results, obtained at the second level, with the contrast invalid > valid conscious trials, to define regions of interest for the subsequent analyses on the unconscious trials. Specifically, we created three spheres (5 mm radius) centered at the peak of activation revealed in the contrast “invalid > valid conscious trials” (see below, the result section). Within these regions of interest, we extracted the beta values resulting from subtracting the absent condition from each condition of interest. These beta values were then used in the following statistical analyses.

Firstly, we wanted to test whether valid and invalid unconscious trials would elicit greater BOLD signal compared with absent trials. To do this analysis, all one-sample *t* tests were one-tailed, as we tested a directional hypothesis. In contrast, comparisons between valid and invalid trials were assessed using two-tailed paired-sample *t* tests, as we did not have a priori predictions regarding the involvement of the ROIs across conditions. For all nonsignificant effects, we additionally report Bayes Factors (BF_01_) to quantify the strength of evidence in favor of the null hypothesis.

## Results

All trials with PAS > 1 were removed from the unconscious (*M* ± SD, 18.4 ± 8.5%) and absent conditions (4.6 ± 8.6%) to ensure no visual experience of the target, and all trials with PAS < 3 were removed from the conscious condition (5.7 ± 7.5%). As a result of this exclusion, the final number of trials included in the analyses was as follows: valid conscious, 51 ± 5 (*M* ± SD); invalid conscious, 23 ± 3; valid unconscious, 102 ± 14; invalid unconscious, 46 ± 7; absent, 40 ± 4.

### Behavioral data

**Figure 2.**
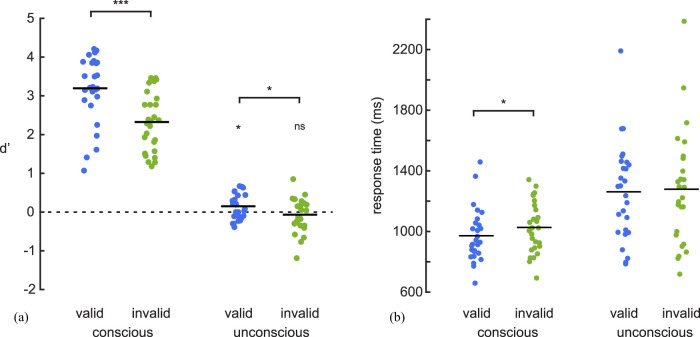
Behavioral results. *D*’ (***a***) and reaction times (***b***) obtained in the conscious and uncoscious working-memory task. ns, not significant. *p < 0.05, ***p < 0.001.

For conscious trials, *d*’ on valid-cue trials was 3.19 ± 0.87 (*M* ± SD) and was significantly reduced for invalid-cue trials (2.32 ± 0.75; *t*_(26)_ = 5.33, *p* < 0.001 one-tailed, Cohen's *d* = 1.03; Fig. 2*a*). Response time was significantly slower for invalid-cue compared with valid-cue trials (invalid-cue: 1,026 ± 170 ms; valid-cue: 972 ± 176 ms; *t*_(26)_ = 2.26, *p* = 0.016 one-tailed, Cohen's *d* = 0.44; Fig. 2*b*).

For unconscious trials, *d*’ on valid-cue trials was significantly better than chance (0.15 ± 0.32, *t*_(26)_ = 2.40, *p* = 0.012 one-tailed, Cohen's *d* = 0.46). Performance was significantly reduced for invalid-cue trials (−0.07 ± 0.43, *t*_(26)_ = −1.87, *p* = 0.036 one-tailed, Cohen's *d* = −0.36) which no longer differed from chance (*t*_(26)_ = −0.81, *p* = 0.79 one-tailed, Cohen's *d* = −0.16, BF_01_ = 8.22; Fig. 2*a*). As a control, we examined whether participants might have treated the distractor presented at the invalid location as the target. To test this possibility, we tested whether the inverse *d*’ [*d*’ = z (false alarm rate) − *z* (hits rate)] was significantly higher than zero for invalid trials. Indeed, since the probe could only match either the target or the distractor, if participants consistently treated the distractor in the invalid condition as a target there would be a higher rate of FA than hits, resulting in a positive *d*’. However, the control analysis revealed no significant difference from zero (0.07 ± 0.43, *t*_(26)_ = 0.82, *p* = 0.21 one-tailed, Cohen's *d* = 0.16, BF_01_ = 2.32). Response time was nominally slower for invalid-cue compared with valid-cue trials, but the difference did not reach statistical significance (invalid-cue: 1,278 ± 371 ms; valid-cue: 1,261 ± 319 ms; *t*_(26)_ = 0.71, *p* = 0.24 one-tailed, Cohen's *d* = 0.136, BF_01_ = 2.61; Fig. 2*b*).

### fMRI data

**Figure 3.**
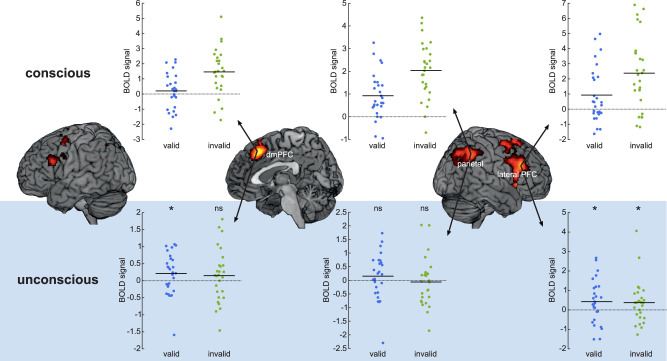
Brain activity during valid and invalid trials. BOLD signal is reported as beta values from the multiple regression analyses for consciously (top part) and unconsciously (bottom part) valid/invalid conditions. Although prefrontal regions were significantly activated for unconscious relative absent trials, there was no significant modulation from cue validity. ns, not significant, *p < 0.05.

Comparing conscious with absent trials, regardless of cue validity, revealed a network of brain regions including occipitotemporal, parietal, and prefrontal cortex, consistent with our previous research using a similar visuospatial task (Pedale et al., 2023). Conscious invalid-cue trials elicited increased BOLD signal relative valid-cue trials in the dorsomedial (dm) PFC, lateral PFC, right anterior insula, bilateral inferior parietal cortex, and frontal eye fields (Table 1, Fig. 3). Previous research and theory on visuospatial (re)orienting of attention suggest that medial and lateral PFC are involved in monitoring and executive control, whereas inferior parietal regions, primarily in the right hemisphere, are crucial for orienting of attention (Petersen and Posner, 2012). We therefore used the results from the conscious trials to define three regions of interest, focusing on the right-sided dmPFC, lateral PFC, and inferior parietal cortex for investigation of unconscious trials (Table 1). Specifically, we created three spheres (5 mm radius) centered at the peak of activation revealed in the conscious > absent contrast (see Table 1 for coordinates).

**Table 1.** Brain regions revealing an effect of validity in conscious trials

Region	MNI coordinates	*t* value	Cluster size
dmPFC	6 22 48	6.57	5,845
Inferior frontal sulcus	50 26 26	5.90	
Anterior insula	32 22 −2	5.49	
FEF	−30 4 46	5.22	
FEF	28 0 46	5.07	
Inferior frontal sulcus	−50 20 24	4.53	
Inferior parietal lobule	44 −46 40	5.44	1,977
Intraparietal sulcus	32 −66 44	4.96	
Intraparietal sulcus	−28 60 48	4.35	470

The table shows the regions that revealed higher BOLD signal during conscious invalid compared with valid trials, with major sub-peaks (indented). MNI, Montreal Neurological Institute.

In the dmPFC, unconscious valid-cue trials were associated with significant BOLD signal change relative absent trials (*t*_(26)_ = 1.81, *p* = 0.041 one-tailed, Cohen's *d* = 0.35; Fig. 3). However, the BOLD signal was no longer significant for invalid-cue trials (*t*_(26)_ = 0.99, *p* = 0.17 one-tailed, Cohen's *d* = 0.19, BF_01_ = 1.91), although there was no significant difference between valid- and invalid-cue trials (*t*_(26)_ = 0.45, *p* = 0.65 two-tailed, Cohen's *d* = 0.087, BF_01_ = 4.46). In the lateral PFC both valid and invalid unconscious trials had significantly higher BOLD signal compared with absent trials (valid: *t*_(26)_ = 1.96, *p* = 0.03, Cohen's *d* = 0.38; invalid: *t*_(26)_ = 1.75, *p* = 0.046, Cohen's *d* = 0.34; one-tailed), with no difference between them (*t*_(26)_ = 0.30, *p* = 0.77 two-tailed, Cohen's *d* = 0.058, BF_01_ = 4.71). In inferior parietal cortex neither valid nor invalid unconscious trials differed from absent trials (valid: *t*_(26)_ = 0.94, *p* = 0.18, Cohen's *d* = 0.18, BF_01_ = 2.02; invalid: *t*_(26)_ = −0.37, *p* = 0.64, Cohen's *d* = −0.072, BF_01_ = 6.37; one-tailed).

## Discussion

Consistent with previous research we found that unconscious valid-cue targets could be remembered across a brief delay to support goal-directed behavior (Gambarota et al., 2022). Furthermore, BOLD signal change in dorsomedial and lateral prefrontal regions was elevated during the encoding of target information, further confirming previous findings of an involvement of prefrontal regions in unconscious working memory (Bergstrom and Eriksson, 2014, 2018; Dutta et al., 2014; King et al., 2016; Pedale et al., 2023). In contrast, we did not find any support for an adaptive deployment of attention toward a target appearing at an unexpected location: BOLD signal in prefrontal regions was not significantly modulated by cue validity and performance dropped to chance level.

For conscious stimuli, invalid-cue trials elicited increased BOLD signal in dmPFC, mirroring previous research where an increased need for cognitive control activates the dmPFC (Shenhav et al., 2016). The current finding that valid-cue unconscious trials elicited significant BOLD signal change in the same brain region is consistent with a study by Dehaene and colleagues, demonstrating increased BOLD signal in dmPFC only for conscious incongruent trials but for both congruent and incongruent unconscious trials (Dehaene et al., 2003). A similar increase for unconscious primes relative conscious primes, but in the lateral PFC, was also observed by Lau and Passingham (2007). Notably, the current and previous findings that unconscious but congruent/valid stimuli elicit activity in cognitive control regions speak against the notion that unconscious processing is “automatic”.

Although dmPFC activity was not significantly modulated by cue validity, BOLD signal was reduced during unconscious invalid-cue trials such that there was no longer a significant difference from absent trials. This pattern is consistent with a report by van Gaal et al. (2008), where PFC activity was seen only when an unconscious stimulus was task relevant. We have previously demonstrated that task relevance can be set only by conscious stimuli (Pedale et al., 2023), which is consistent with the current findings. That is, the conscious cue sets task relevance in term of shape and indicates, with high probability, the left/right (though not vertical) position of the target. An unconscious change in task-relevant position (i.e., invalid-cue trials) did not elicit a change in attentional deployment. Nevertheless, activity in lateral PFC remained significant also for unconscious invalid-cue trials.

The reorientation of attention required for successful performance on invalid-cue trials suggests an involvement of the ventral attention network, including the temporoparietal junction (TPJ; Corbetta and Shulman, 2011; Petersen and Posner, 2012). Indeed, conscious invalid-cue trials were associated with increased BOLD signal in inferior parietal and prefrontal regions, although the locus of the inferior parietal activation was here more dorsal than typical TPJ coordinates (Geng and Vossel, 2013). However, there was little evidence for parietal involvement during unconscious trials, consistent with the apparent lack of a reorienting response during unconscious invalid-cue trials.

This pattern of results is in line with the well-recognized time course of anterior-to-posterior brain activation for top-down attention versus posterior-to-anterior bottom-up attention (for a review, see Katsuki and Constantinidis, 2014). In the current design, the cue initiates top-down attention toward a goal-defined shape but also promotes spatial attention to one of the sides of the screen. Top-down attention has been extensively demonstrated to be initiated by anterior brain regions with subsequent activation of posterior parietal regions. For example, animal studies revealed that a visual search task starts with the firing of neurons in the prefrontal cortex, followed by neurons in the posterior parietal cortex (Buschman and Miller, 2007; see also Ibos et al., 2013 for similar findings). Here, coherent with this time course, we revealed an activation in the lateral and medial portion of the PFC for unconscious valid stimuli, suggesting its role in signaling the need for cognitive control operations. However, this activation seems to be too weak to initiate the subsequent posterior activation. What is more, in the invalid condition the signal—still detectable in dlPFC—is not significant in the dmPFC, thus providing insight into different neural processes triggered by cue validity.

To interpret this result, it is important to note that also in the invalid condition a (nontarget) shape is present in the spatially attended location. The lack of adaptive attentional deployment to the other side in the invalid condition may reflect two possibilities: either participants had extracted information that the shape on the cued side did not match the target, but this was insufficient to initiate an attentional shift; or they were unable to extract shape information and thus defaulted to treating all shapes at the cued location as valid. However, when comparing false alarms to hits, no such bias emerged, suggesting that responses were not based solely on the conscious cue. This indicates some processing of the mismatch, albeit insufficient to support effective attentional reorienting (see also Pedale et al., 2023, for similar evidence of above-chance discrimination between target and distractor). Moreover, as suggested by Gayet et al. (2020) spatial attention is not a sufficient condition for promoting enhanced processing of all stimuli presented at the attended location. In agreement with this view, valid stimuli—matching the cue shape—may be enhanced and processed by dm/dl PFC leading to better than chance performance. On the contrary, invalid stimuli trigger a reduced dmPFC brain activation, but such initial brain “intuition” of an invalid condition is too subtle to produce an attentional shift to the other side of the screen. A lack of reactivity in parietal regions for unconscious stimuli in general (i.e., for valid and invalid stimuli) does not allow the required “circuit breaker” to redirect attention to the opposite target location. This is consistent with a view of bottom-up attention as directed by salient stimuli, which is by definition absent in the unconscious condition.

Bottom-up attention appears to begin in the parietal cortex, with subsequent activation of PFC (Buschman and Miller, 2007). fMRI studies contrasting valid versus invalid spatial cues have extensively revealed activation of ventral frontoparietal areas (e.g., the right TPJ) and bilateral intraparietal sulcus (Vossel et al., 2006, 2012; Corbetta and Shulman, 2011) as in the current invalid conscious condition. This ventral activation could signal the violation of top-down expectations in the invalid condition to the dorsal attentional network, which would be responsible for the reorientation to the opposite target location. Taken together, the unconscious invalid target is not salient, so is unable to initiate the bottom-up process. Thus, the entire reorientation mechanism is to be carried out by the frontoparietal top-down attentional network, which, as described above, appeared to be not enough to initiate the required attentional shift. In summary, we may interpret the above set of findings in the following way: dmPFC signals the need for cognitive control operations but dwindles in amplitude when there is a mismatch between information regarding target shape and position. A lack of reactivity in ventral parietal regions (in the vicinity of the TPJ) for unconscious stimuli prohibits the needed “circuit breaker” function to redirect attention to the actual target location. A similar phenomenon was observed by Fuchs and Ansorge (2012), who reported sustained attentional facilitation at the location of an unconscious cue even at long stimulus onset asynchronies (SOAs), in the absence of inhibition of return. Although their cues were unconscious and targets conscious (i.e., the reverse of our design), both results suggest that attention guided by unconscious processing lacks flexibility and does not effectively engage reorienting mechanisms.

Finally, some important caveats should be considered when interpreting these results. In the present study, we employed a subjective measure to assess conscious experience (i.e., PAS). We chose it because, although subjective measures may risk overestimating unconscious processing due to a conservative response bias, they provide a measure of consciousness at the trial level. In the current experiment, the number of absent trials reported as seen (PAS > 1) indicates that participants had a liberal response bias (i.e., they are likely to report that they did see something even though there was no stimulus presented). Consistent with this behavior, the PAS has been shown to be more sensitive and exhaustive than alternative subjective methods (Sandberg et al., 2010) such as confidence ratings (Cheesman and Merikle, 1986) or postdecision wagering (Persaud et al., 2007). In contrast, objective measures have low face validity and risk underestimating unconscious effects by misclassifying unconscious processing as conscious (Merikle et al., 2001). While our choice of PAS is motivated, its potential limitations should still be acknowledged when interpreting the present findings. The second issue regards multiple-comparison correction: it should be noted that the ROI-based results would not survive multiple-comparisons correction. Nevertheless, we believe that they remain relevant and critical to the interpretation of unconscious attention selection, given the expected subtlety of unconscious effects and the inherent difficulty in detecting weak neural activations associated with nonconscious processing.

Notwithstanding these limitations, our interpretation of the current results is that prefrontal activity increase may indicate an attempt at working memory, but which leads to failure in certain conditions, e.g., when attention needs redirection to deal with unexpected situations. That is, while the findings are consistent with the existence of certain aspects of unconscious working memory (i.e., cognitive control operations that link current stimuli to goal representations), they do not support the flexible control of attention required for “good” working memory.

## Synthesis

Reviewing Editor: Ifat Levy, Yale School of Medicine Decisions are customarily a result of the Reviewing Editor and the peer reviewers coming together and discussing their recommendations until a consensus is reached. When revisions are invited, a fact-based synthesis statement explaining their decision and outlining what is needed to prepare a revision will be listed below. The following reviewer(s) agreed to reveal their identity: Blaire Dube. Note: If this manuscript was transferred from JNeurosci and a decision was made to accept the manuscript without peer review, a brief statement to this effect will instead be what is listed below.

The manuscript provides an important step forward in distinguishing functional differences between consciously and subconsciously perceived stimuli. The authors have addressed all initial concerns, and the additional analysis is particularly convincing.
